# Effects of Psychological Stress on Innate Immunity and Metabolism in Humans: A Systematic Analysis

**DOI:** 10.1371/journal.pone.0043232

**Published:** 2012-09-19

**Authors:** Sushri Priyadarshini, Palok Aich

**Affiliations:** School of Biological Sciences, National Institute of Science Education and Research (NISER), Institute of Physics Campus, Sachivalaya Marg, Bhubaneswar, Odisha, India; University of Jaén, Spain

## Abstract

Stress is perhaps easiest to conceptualize as a process which allows an organism to accommodate for the demands of its environment such that it can adapt to the prevailing set of conditions. Psychological stress is an important component with the potential to affect physiology adversely as has become evident from various studies in the area. Although these studies have established numerous effects of psychological stress on physiology, a global strategy for the correlation of these effects has yet to begin. Our comparative and systematic analysis of the published literature has unraveled certain interesting molecular mechanisms as clues to account for some of the observed effects of psychological stress on human physiology. In this study, we attempt to understand initial phase of the physiological response to psychological stress by analyzing interactions between innate immunity and metabolism at systems level by analyzing the data available in the literature. In light of our gene association-networks and enrichment analysis we have identified candidate genes and molecular systems which might have some associative role in affecting psychological stress response system or even producing some of the observed terminal effects (such as the associated physiological disorders). In addition to the already accepted role of psychological stress as a perturbation that can disrupt physiological homeostasis, we speculate that it is potentially capable of causing deviation of certain biological processes from their basal level activity after which they can return back to their basal tones once the effects of stress diminish. Based on the derived inferences of our comparative analysis, we have proposed a probabilistic mechanism for how psychological stress could affect physiology such that these adaptive deviations are sometimes not able to bounce back to their original basal tones, and thus increase physiological susceptibility to metabolic and immune imbalance.

## Introduction

Physiological homeostasis is a dynamic equilibrium of the body maintained by the interplay of various biological processes and interactions with the environment. When this state of dynamic equilibrium is perturbed, adaptive responses try to overcome threats from the challenge with the goal of restoring the original state [1]. Stimuli that perturb homeostasis can be considered as stressors that affects physiology and can make individuals susceptible to diseases [2], [3]. Although there are reports which correlate stress with disease susceptibility [4], [5], [6], [7], [8], a systematic analysis is required in order to understand the molecular and genetic basis of such correlation as well as to develop a strategy for future work in establishing the predictability of stress-induced disease susceptibility. This view is supported by the fact that the traditional notions about the immune system as a closed system have evolved, the recent views being that there exists multi-directional communication among various physiological processes namely, immunity, metabolism and neurological signaling [9]. Under the influence of stressors, specific molecules and cells are produced as a requirement for activation of immune and other physiological processes, which in turn demands high energy utilization for rebalancing of physiological homeostasis. Such energy requirements may cause global alterations at the systems level (such as a shift in the energy balance) in order to maintain homeostasis. It is important to mention here that the increased and altered energy demands affect various physiological processes of which metabolism and immunity are two critical components in maintaining health.

Psychological stress affects physiological energy balance via activation of the Hypothalamic-Pituitary-Adrenocortical (HPA) and/or the Sympathetic-Adreno-Medullary (SAM) axes by producing different stress hormones such as cortisol, epinephrine and norepinephrine [10], [11], [12]. Energy utilization and balance can also be modulated by alterations in many other processes during the stress response, for example, feeding behavior [13], [14], metabolism [15], [16] and reproductive behavior [14], [16], [17]. It is reported that the endocrine system together with the nervous system might play a significant role in the modulation of metabolism and energy distribution [18]. These studies have established that it is the inflammatory innate immune responses that is activated first when the body is subjected to psychological stress [19]. There are also indications that innate immunity and acute phase responses are integrated with the neuro-endocrine system [20]. Hence, we believe that there should be a very sophisticated mechanism by which communication among these processes would regulate homeostasis, making it possible for the physiology to perceive and accommodate fluctuations in its internal environment during the adaptive response to psychological stress such that physiologic integrity is maintained. In an attempt to understand this delicate balance among various physiological processes, we bagan with an analysis of the cross-talk between innate immunity and metabolism which, based on the above arguments, should be important frontiers for any adaptive response to psychological stress.

There are numerous isolated investigations reporting complex data regarding genes, pathways and networks affecting innate immunity [21], [22], metabolism [23], [24] and psychological stress [25], [26]. Most of the investigations carried out so far have measured aspects of immunity, metabolism or other processes in relation to psychological stress in an isolated manner. However, studies attempting to correlate psychological stress with physiological processes such as innate immunity, metabolism and their relationship at the genomic and molecular levels are scarce [27]. It is, therefore, important not only to identify candidate genes associated with adaptive response to stress, but also to understand the gene networking to determine impact on physiology by analyzing the molecular and causative functional relationships among apparently unrelated candidate genes. The current report has (a) analyzed results existing in this area, and (b) extended biological information obtained from various studies reported so far. The current approach has used gene-association networks in an attempt to visualize a more global picture in order to better understand the effects of psychological stress and the maintaining of balance between metabolic and innate-immune processes in humans. This work will provide a basis and better define a direction for future research in the area.

Over last few years, information concerning the human interactome has grown rapidly (e.g. KEGG, HPRD, BIND, MINT, miRBase, BIOMART databases, to name but a few). However, the data still remains largely incomplete lacking systematic information about connectivity at molecular level [27], [28], [29]. Many other tools also exist that can identify candidate genes for a particular phenotype by mining the available interactome data (e.g. Endeavour, SUSPECTS, GeneSeeker, PROSPECTR) [25], [30], but they do not provide the required gene networking which is essential in understanding the relationships involved in a complex response. Databases like STRING [31], which use known and predicted protein-protein interaction data, do provide gene networks based on the identified candidate genes. However, they lack robust user-controlled query systems which are required for association of genes to the phenotypes under study. Network-based methodologies have been developed for the prediction of genes associated with diseases [32], [33], however, these are limited by lack of exhaustive disease-gene annotation for many diseases in OMIM [34], [35], [36]. A few recently developed methods [37] exist that identify gene-phenotype associations using BIOMART instead of gene-disease association for pulling out the causative genes and associating them with the phenotypes. Despite these improvements in the available tools, developing a systematic understanding of data connectivity is still only partial. This is due to the fact that all disease phenotypes are not completely annotated and many are yet to be listed in OMIM, on which the learning framework of these tools largely depends. To address the above challenges, literature based data-sets have also been used [38], [39], [40] as an aid since they are much richer in interaction data compared to the annotated databases. Text-mining, if used solely for identifying candidate genes and their phenotype associations, might induce bias towards more explored diseases and phenotypes. Moreover, text-mining alone will not be able to identify all functionally related genes if reports are absent in the literature, which can be overcome by data-mining of bio-ontology databases [41], [42].

We have, therefore, used a strategy (Schema S1) that establishes connectivity in the information scattered across the pertinent literature through an unique meta-analysis approach following four major steps: 1) Identifying candidate genes for metabolism and innate immunity based on training genes which have already been established to be associated with those processes from the human interactome by data-mining, 2) Screening for associations of candidate genes, within the biological contexts of interest, using user controlled context-based information in the published literature, 3) Building of an association among the elements of the context-dependent screened data and 4) Enrichment analysis of the screened data to form associations for evident cues on the relationship between innate immunity and metabolism in the context of psychological stress. The current report helps our understanding of the inter-dependence and balance between innate immune system and metabolic processes, when perturbed by psychological stress, at systems level. The uniqueness of the current study lies not in the use of network-based approaches, but in using the existing tools for candidate gene identification and network generation. This approach overcomes the limitations of the existing methods and increases the confidence level of predictions based on these methodologies (see materials and methods). The networks thus established can help to develop a preliminary understanding of biologically relevant information in order to identify important patterns hitherto hidden and subsequently help us in designing effective future strategies to understand stress dependent disease susceptibility. Information in the current report could also be used for elucidating, in part or in whole, any possible predictive mechanisms which would serve a critical role, in answering key biological questions pertaining to human physiology.

## Methods

**Figure 1:**
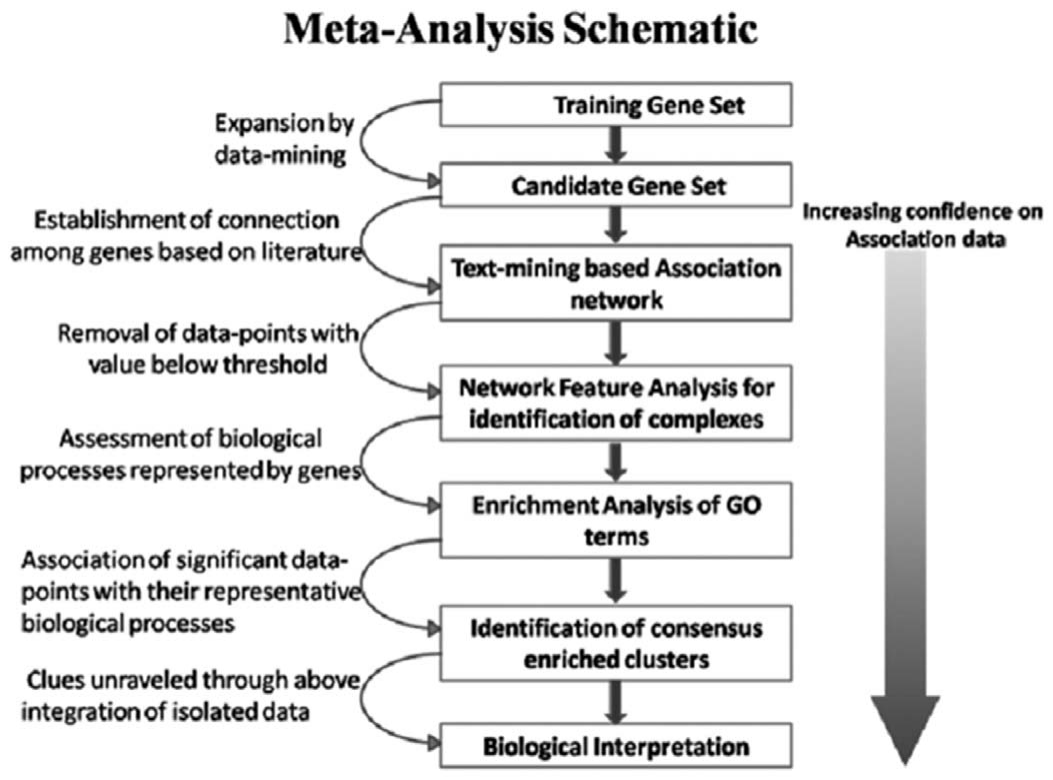
Methodological schema. The step-by-step schematic of the approach developed to identify functional modules that could be used to understand the relationship between innate immunity and metabolism in normal physiological conditions as well as in relation to psychological stress.

In the absence of high throughput data in the context of psychological stress and the afore-mentioned physiological processes, we decided to analyze existing data in the literature through data-mining and text mining to create gene-association networks for identifying the genes associated with innate immunity, metabolism and psychological stress. Efficient screening of data requires extracting data from both structured repositories (databases) as well as from unstructured textual documents. Powerful exploratory techniques like data-mining are used for automatic discovery of patterns from structured databases. For handling unstructured data, text-mining comes in handy since it uses language-based techniques to parse textual data and identify patterns. A number of data-mining and text-mining tools exist that cover different aspects of information extraction from databases and from biomedical literature in a variety of domains but, none of these are customized for an integrated approach that combines both mining techniques to extract information in relation to specific contexts and, at the same time, build associations from the extracted terms. We have taken a unique dual approach in combining data-mining and text-mining techniques which could overcome their individual short-comings and at the same time build associations among these screened elements, thereby forming association networks. Drawing any biologically relevant information from these networks requires a detailed analysis which could help us identify hidden patterns in the huge data-sets created by the above mentioned mining techniques. We have focused on a bi-facetted analysis of the networks in 1) identifying biological modules (complexes with high relative significant local density as compared to a less dense background) through topological distinctions by analyzing the gene association network structure and 2) carrying out an enrichment analysis for identification of over-represented Gene Ontology terms. This is then followed by an analysis which identifies any correlation between these modules and the over-represented GO-terms. The overall schematic for the approach developed is presented in figure 1.

### Searching and Selection

#### Data-mining

Since, there are no extensive direct studies which explore the relationship between innate immunity and metabolism, we used an indirect association-based approach which sorts data to build a model based on the annotation profile of training-set genes that have established associations with each of these processes. The annotation profile obtained from this model was then used to pull out a list of candidate genes from a defined background. This approach is based on the extrapolation of annotation profiles of genes associated with similar biological function or those involved in the same pathways due to their regulation and expression [43]. Endeavour, freely available at http://homes.esat.kuleuven.be/~bioiuser/endeavour/index.php, was used for identifying the candidate genes (see Supplementary Information) from the whole human genome based on their similarity/association with the “training set”. Top ranking candidate genes were then used for text mining and association network generation.

#### Association network generation through text-mining

To further ensure that the candidate genes were actually biologically related to the processes we aimed to analyze, we used an approach that could query published literature in relation to the context, and at the same time build an association network based on the text-mining results. This added more robustness to our data by serving as an additional step to filter out any unrelated genes from the candidate gene sets that might have been pulled in because of possible algorithmic limitations. Additionally, it allowed extension of the scope of analysis by pulling in those genes into the network, which have a high degree of association with identified candidate genes. The Agilent Literature Search plug-in contained in Cytoscape was used for text-mining literature using structured queries and for generating gene-association networks based on the text-mining results. Four primary gene association networks were generated using four different query structures. These networks included: networks for genes associated with innate immunity “**II**”, genes associated with innate immunity and psychological stress “**II(S)**”, genes associated with metabolism “**M**”, and genes associated with metabolism and psychological stress “**M(S)**”. The literature search plug-in allows the user to control the search options, and provides for custom-made query construction so that the biological relevance of the context can be tightly regulated. Biologically relevant contexts were provided as context terms in the context panel, while the genes from the candidate gene-set were used as search terms. Text mining for each query set is done using PubMed search engines for fetching literature documents which are then parsed into sentences. A lexicon set is used for defining gene names (concepts) and interaction terms (verbs) of interest. The parsed sentences are analyzed for known interaction terms and an association is extracted for every sentence containing at least two concepts and one verb. The concepts and verbs represent the nodes and edges respectively on the Cytoscape canvas [44]. The query structures thus built were as follows:

#### Innate immunity

Gene name AND ((“innate immunity” AND (Homo sapiens OR human)) OR (inflammation AND (Homo sapiens OR human))).

#### Metabolism

Gene name AND ((“metabolism” AND (Homo sapiens OR human)) OR (“metabolic syndrome” AND (Homo sapiens OR human))).

#### Innate immunity and psychological stress

Gene name AND ((“innate immunity” AND “psychological stress” AND (Homo sapiens OR human)) OR (inflammation AND “psychological stress” AND (Homo sapiens OR human))).

#### Metabolism and psychological stress

Gene name AND ((“metabolism” AND “psychological stress” AND (Homo sapiens OR human)) OR (“metabolic syndrome” AND “psychological stress” AND (Homo sapiens OR human))).

The maximum engine match limit was set to 100 for ‘**II**’ and ‘**M**’, while for ‘**II(s)**’ and ‘**M(s)**’ it was set to 500. Maximum engine matches is the number of articles that are searched for each query. The concept lexicon was limited to *Homo sapiens*, so that only articles containing studies or data related to humans would be pulled out.

#### Validity assessment

The text-mining strategy shapes the confidence of the association network data, since it determines the nature and number of literature references that are used for building the associations. Although it reduces bias in such studies, publication bias might still skew the interpretations towards the more widely studied areas. This risk has been overcome to some extent by limiting the maximum number of searches per query to an optimal number such that the evidence for less studied areas is not masked by those of the well studied ones and also through careful choice of context. The confidence on each of these text-mining based association networks was calculated as follows:
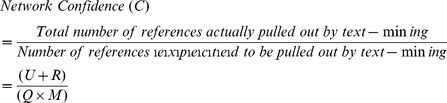
Where,

**U  =  Number of unique references drawn,**

**R  =  Number of redundant references drawn,**

**Q  =  Number of queries used in the text-mining and**

**M  =  Maximum number of references searched per query.**

#### Data abstraction

Understanding the biological relationship between innate-immunity and metabolism in the absence and presence of psychological stress require analysis of networks. Therefore, first four primary association networks were used for generation of more networks as intersection and/or union function of these networks. The derived networks can be described as follows:

**II
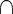
M:** Intersection of innate immunity and metabolism network

**II(S)
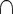
M(S):** Intersection of innate immunity (stress) and metabolism (stress) network

**II
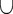
M:** Union of innate immunity and metabolism network

**II(S)
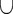
M(S):** Union of innate immunity (stress) and metabolism (stress) network

**Advanced Network Merge plug-in** was used in Cytoscape for generation of networks as union or intersection derivates of these primary networks. The force-directed layout tends to expose the inherent structure of the network in way that facilitates identification of clusters of tightly connected nodes which suggest functional modules and hub nodes which have many interactions and mainly represent functionally important genes. But, it does not eliminate unconnected or sparsely connected nodes that might lead to many false positive predictions. Hence, **MCODE (Molecular Complex Detection) plug-in** in Cytoscape was used which allows retention of an edge in the network only if the given edge is among the highest scoring candidate edges for both genes [45]. This is referred to as the “top overlap” method and it helps in overcoming the issue of false positives. The significant modules, that is modules with MCODE score more than 2.5 and a minimum of 4 nodes (numbers shown in Table S3), were then further analyzed for functional annotation.

#### Quantitative data synthesis

Network Analysis plug-in was used for measuring the node degree (the number of connections of a node) and the clustering co-efficient (degree involvement of a node in the participating clusters) of the nodes in these networks after applying a clustering algorithm to them. It has provisions for visual representation of node degree and relationship to function. Networks thus generated indicate the associations between genes and their degree involvement in the participating clusters, thereby guiding in the singular functional annotation but, does not however give a lead in extracting biological processes, such as metabolic pathways, immune-activation processes, and stress responses.

Gene Ontology enrichment anaLysis and visuaLizAtion(GORILLA) is freely available at the website http://cbl-gorilla.cs.technion.ac.il/. It was used for enrichment analysis to interpret the behavior of one or a combination of clusters or even the whole network such that meaningful biological clues could be identified suggestive of possible mechanisms elucidating the role of various biological processes [46]. Comparison of the genes associated with metabolism, innate-immunity and psychological stress with a background consisting of the whole genome would give a wrong representation of the involvement of these genes in psychological stress in terms of Gene Ontology enrichment. So, innate immunity and metabolism genes associated with psychological stress were compared against a background that consisted of a complete set of genes associated with innate immunity and/or metabolism irrespective of their association with psychological stress. GO terms with a minimum enrichment p-value of 10^−7^ were included in the analysis.

## Results

### Identification of Candidate Genes and Generation of Association Network

**Figure 2:**
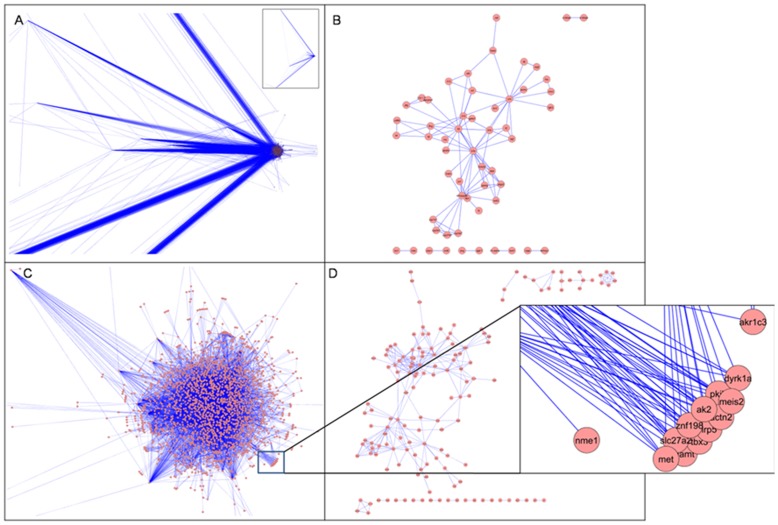
Primary text-mining association network topologies. Network topologies (after applying force-directed layout) of the text-mining association networks obtained by using the Agilent literature search plug-in have been shown here. The networks consist of genes associated with A) Innate Immunity, B) Innate immunity and psychological stress, C) Metabolism and D) Metabolism and psychological stress. Four different query structures were used to generate A, B, C and D.

**Figure 3:**
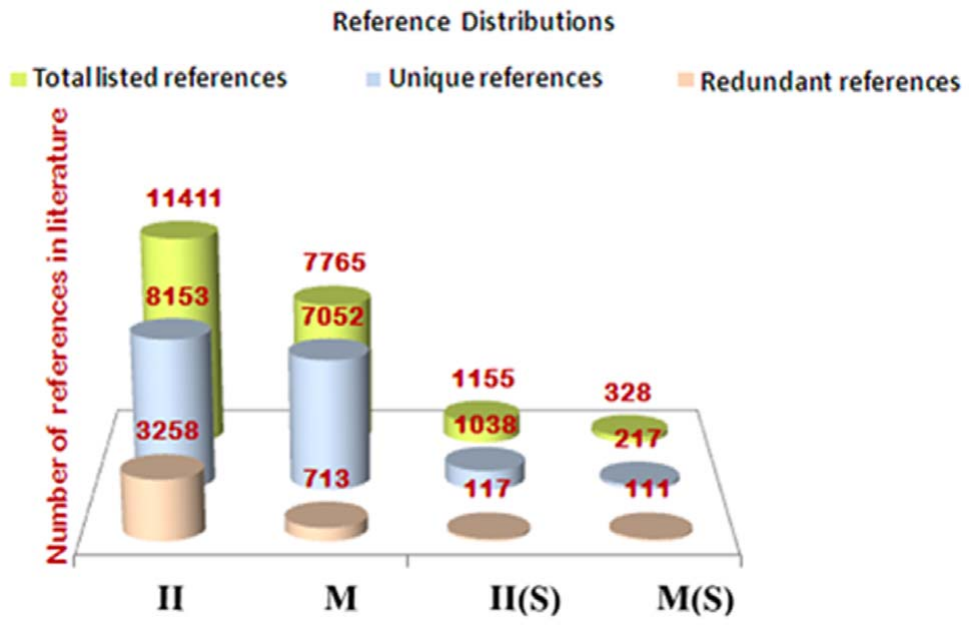
Literature reference distribution. The distribution of references, used by the Agilent literature search plug-in for generation of the four primary networks, is shown. The references that have been used more than once for extracting associations are redundant while those used just once are unique.

The top 100 and 200 genes, from the candidate gene set for innate immunity and metabolism, respectively, were used in the generation of text-mining based association networks II and M, respectively. The list of training-set genes for metabolism and innate-immunity as well as the candidate genes thus pulled out for each training set are provided in the supplement (Tables S1 & S2). Text-mining association networks thus generated, do not contain experimentally determined interactions. Rather, these have more general association types and offer an alternative network source where interaction data are limited [44]. A view to the topologies of the first four primary association networks is shown in figure 2 and their network statistics are given in Table 1. As can be seen from the reference distribution shown in figure 3, the network confidence (C) for **M(S)** [metabolism and psychological stress] and **II(S)** [innate immunity and psychological stress] networks is much lower compared to that of the **M** [metabolism] and **II** [innate immunity] networks, respectively. This is due to the keyword “psychological stress” that is used as an additional filter which indicates that far fewer studies have been done on psychological stress in relation to innate immunity and metabolism. This, together with the assessment of the screened references revealed that keyword search-based text-mining did not draw non-contextual data-points and thus this helps validate our methodology. It was interesting to note that even though network **II** contained fewer nodes compared to the **M** network, the number of edges is more for the **II** network. This indicates that either the nodes representing genes in the **II** network are multifunctional, or that there is more literature present in relation to the nodes in network **II**. Since the unique reference distribution does not reflect significant differences between the **II** and **M** networks (**II**-8153, **M**-7052), the chances that genes which have been so far associated with innate immune functions of being multi-functional is more likely. Tables S5–S10 describe the detailed contributions of the literature references as well as for nodes and edges.

**Table 1:** Details of reference distribution and network statistics of the four primary association networks.

	Reference distributions				Topology feature details				
Network	Total references	Unique references	Duplicate references	Network Confidence	Nodes	Edges	No. of isolated nodes	Avg. num. of neighbors	Clustering co-efficient
**Metabolism, M**	7765	7052	713	0.7059	2108	9833	0	9.329	0.528
**Innate Immunity, II**	11411	8153	3258	0.5763	1721	13989	0	16.257	0.581
**Metabolism & Stress,** **M(S)**	328	217	111	0.0298	148	314	0	4.243	0.452
**Innate Immunity &** **Stress, II(S)**	1155	1038	117	0.0586	55	102	0	3.709	0.476

The table contains details of the first four primary association networks obtained by text-mining results using Agilent Literature search (ALS) plug in. ‘Total references’ refers to the total number of literature references used by the ALS plugin to generate the networks, ‘Unique references’ refer to the number of references that were used only once for the network generation while ‘Duplicate references’ refer to the number of references used more than once for network generation. ‘Network clustering coefficient’ is the average of the clustering coefficients for all nodes in the network and ‘average number of neighbors’ indicates the average connectivity of a node in the network.

### Network Analysis

**Figure 4:**
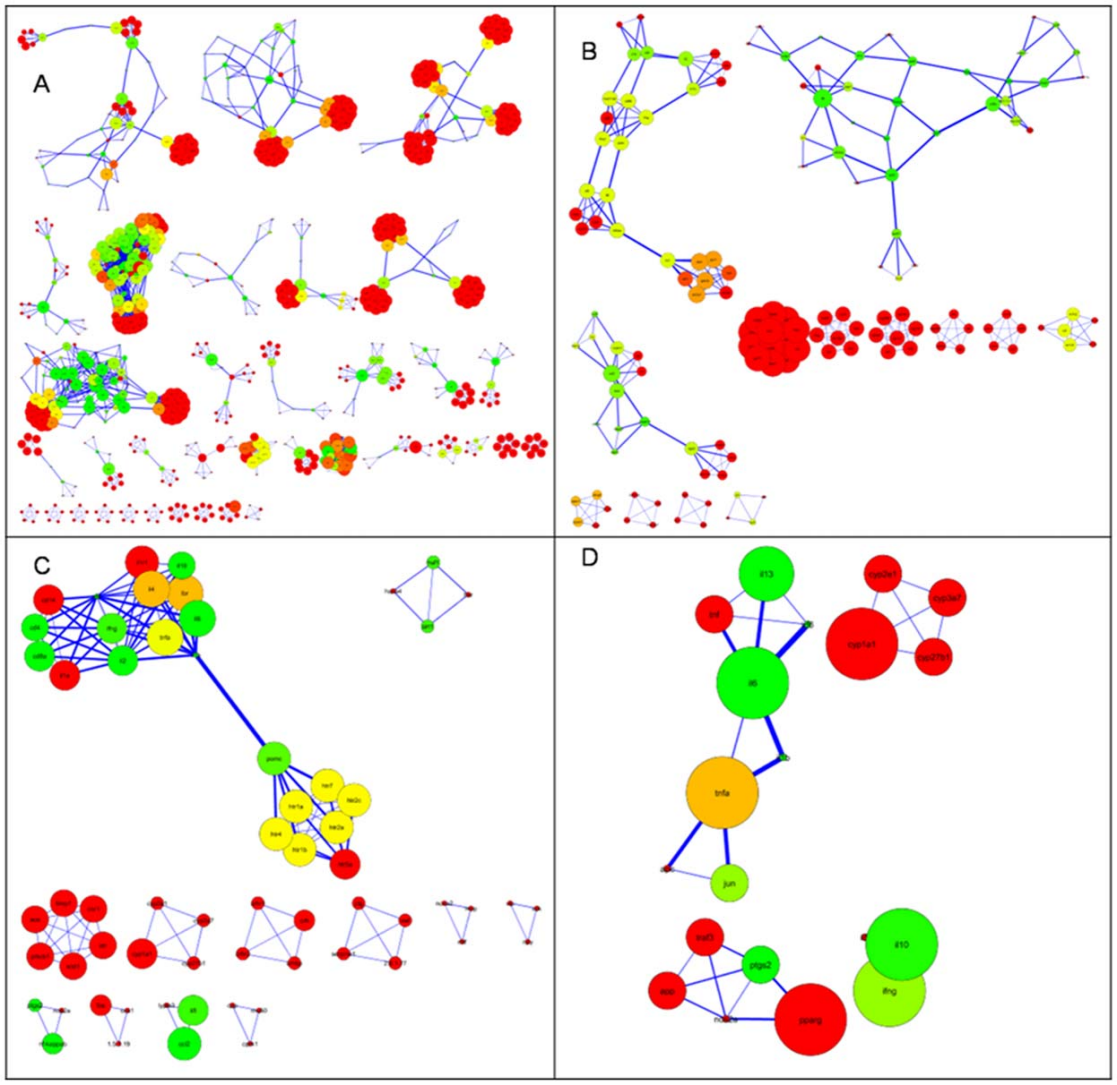
Derived association network topologies. Network topologies are shown for the derived networks after applying clustering algorithms using MCODE. A-II
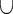
M network containing clusters with a minimum MODE score of 1.5, B- II
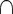
M network containing clusters with a minimum MCODE score of 1.25, C- II(S)
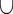
M(S) and D- II(S)
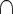
M(S) networks containing all clusters. Larger nodes represent higher node degree, while warmer colors represent higher clustering co-efficient, where warmer to cooler colors are represented by a gradient from Red to Green.

**Figure 5:**
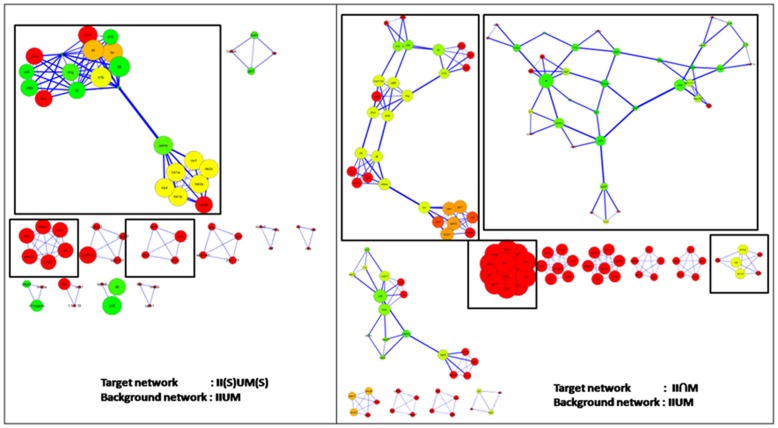
Consensus enriched clusters. Networks showing the consensus clusters after enrichment analysis. The Consensus clusters are shown in boxes in the networks II(S)
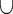
M(S) and II
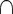
M.

**Figure 6:**
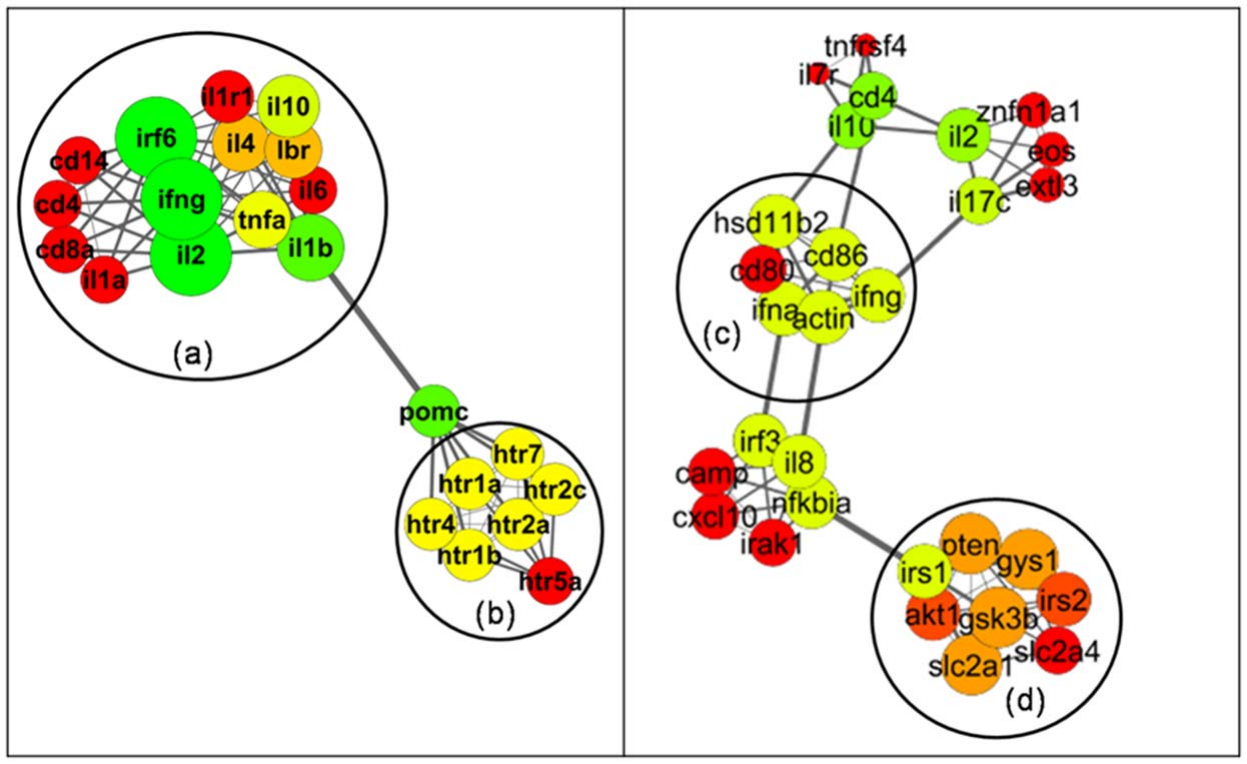
Sub-cluster identification and analysis from enriched clusters. Cluster 4 from the II
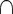
M and Cluster 10 from the II(S) 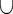
M(S) networks are shown here. Various sub-clusters are shown for (a) inflammatory cytokines, (b) serotonin receptors, (c) genes involved in inflammation and metabolism and (d) genes involved in lymphocyte activation as well as positive regulation of catabolic processes.

Identification of the modules provided another level of functional annotation above the guilt-by-association methods used for generation of text-mining based association networks [45] [47], where, any false positive association pulled out could lead to faulty functional annotation of those genes. Figure 4 shows the topologies of the derived networks (i.e. **II
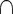
M**, **II(S)
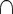
M(S)**, **II
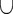
M** and **II(S)
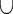
M(S))** after applying clustering algorithms and normalizing for proper representation of node degree and clustering-coefficient in these networks (see methods) [original Cytoscape cluster data are provided as supplementary files in PSI-MI XML format, see PSI-MIData S1]. It can be seen that data points with lower confidence have been removed, leaving only those nodes and edges that participate in cluster formation (their retention is owing to their MCODE 4 high scores) for functional analysis. Table 2 details the numbers of nodes, edges and clusters in each network after clustering and normalization to significant values. Enrichment analysis of **II
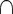
M** against the **II
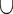
M** background showed the GO terms enriched due to genes that are associated both with innate immunity and metabolism, irrespective of their association with psychological stress. However, genes that have been extensively implicated in relation to psychological stress and lack enough evidence for their association with innate immunity and metabolism would not be presented in the enriched terms. This situation called for an enrichment analysis of **II(S)
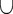
M(S)** against the **II
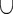
M** background to identify such genes and it revealed that among all other genes CNR1, HTR1B, HTR2A, CCL2 were consistently present in most of the enriched terms. Enrichment analysis of **II
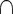
M** (enriched GO biological processes represent genes involved in regulating both innate-immunity and metabolism only) and **II(S)
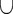
M(S)** (enriched GO biological processes represent genes common to both innate immunity and metabolism and at the same time are associated with psychological stress) against a background of **II
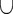
M** revealed certain specific consensus clusters that were consistently enriched (shown in figure 5). The details of the enrichment terms and genes associated with these clusters are shown in Table S3 (details of the complete set of enrichment terms have been included in the supplement in PSI-MI XML format, see PSI-MIData S1). Detailed analysis of these consensus enriched clusters highlighted tight sub-clusters populated with high MCODE score-bearing genes within select few clusters as shown in figure 6. It was seen that in cluster 1 of the **II(S)
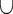
M(S)** network (refer to Table S3), a tight sub-cluster was formed by the genes coding for inflammatory cytokines (marked as ‘**a**’), which was connected to another sub-cluster formed by the 5-HT receptor family genes (marked as ‘**b**’) via the POMC gene. Similarly, GSK3, SLC2A1, AKT1, IRS1, IRS2, PTEN and GYS1 formed a tight sub-cluster (marked as ‘**d**’), while HSD11b2, IFNa1, ACTIN, CD80, CD86 and IFNg formed another tight sub-cluster (marked as ‘**c**’) in cluster 4 of the **II
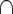
M** network (refer to Table S3). A detailed study of the annotation profiles of these sub-clusters provided important basis for predictive assignment of function to individual relevant genes which will be discussed in a later section. Enrichment analysis of **II(S)
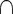
M(S)** against a background of **II
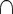
M** and **II
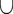
M**, however, resulted in very few clusters and these had very low MCODE scores due to lower number of genes in the **II(S)
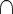
M(S)** network. Details of the significantly enriched terms (only those GO terms with a minimum enrichment p-value of 10^−7^ have been included in the functional analysis) obtained in the enrichment analysis of **II(S)
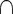
M(S)** against a background of **II
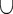
M** case have been provided in Table S4.

**Table 2:** Network statistics of the derived networks reflecting the effect of clustering.

Network	Nodes		Edges		Clusters
	Before clustering	After clustering	Before clustering	After clustering	
II∪M	2904	639	22581	3413	30
II(S)∪M(S)	158	562	341	143	7
II∩M	925	89	1241	265	8
II(S)∩M(S)	45	20	75	29	4

The networks derived as union functions have been allowed to retain clusters with minimum MCODE score 1.25, while all the clusters have been retained in the networks derived as intersection functions (see materials and methods).

### Predictive Assignment of Function using Annotation Profile of Sub-clusters

The node-degree is defined as the number of edges from a node, while the average clustering co-efficient is the tendency of a node to form a cluster in a network. A gene connected with more than one associative gene would be expected to exhibit a higher node-degree and therefore high node-degree correlates with a possible multi-functional role of the gene. Again, the genes of a module are usually associated with the same biological function. Therefore, a gene having a high clustering coefficient in a module can be assigned a predictive function in the direction of the functional profile of that module. The overall functional profile of the genes from sub-cluster ‘**d**’ (figure 6) in cluster 4 of the **II
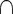
M** network indicates their involvement in lymphocyte activation (namely inflammation) [48] as well as positive regulation of catabolic activities (glycogen and glucose breakdown) for release of energy. However, the enrichment analysis showed that GSK3b and GYS1 are the two genes not present in the significantly enriched GO terms, indicating that the role of these two genes in both of the above mentioned areas has not been well established. Interestingly, the association network data revealed that both of these genes have high MCODE scores (2.370 each) and the highest node degrees (7 each) within the sub-cluster, which indicates their high degree of contribution to the sub-cluster. On reviewing the literature, it was found that GSK3b has been implicated in neuro-inflammation in addition to its reported role in glycogen metabolism which matches with the functional profile of the sub-cluster, thereby confirming the efficiency of this unique functional annotation strategy. GYS1 (Glycogen Synthetase 1) has been implicated in the storage of muscle glycogen that provides critical energy during bursts of activity and sustained muscle exertion but there have been no direct reports of its involvement in inflammation or cell activation as of yet. Following the principles of functional profile similarity in the sub-cluster, we can, therefore, also assign a predictive involvement of the GYS1 gene during inflammation. Similarly, in the same cluster the functional profile of another sub-cluster ‘**c**’ shows the role of involved genes in inflammation and lipid metabolism. By the same principles as for sub-cluster ‘d’, IFNa1 which is already known to be involved in inflammation (but not many reports of its involvement in the regulation of metabolism are present), can be predicted to have an association with lipid metabolism. Again HSD11b2, reported to be involved in lipid metabolism and carbohydrate metabolism but not with inflammation, can be assigned a predictive function associated with inflammation. These genes need not have a direct involvement in predicted functions like lymphocyte activation, inflammation or lipid and carbohydrate metabolism. They might indirectly contribute to regulation of these prediction-based assigned functions, which then need to be established by experimental evidences. Our analysis throws light on the most probable as yet unexplored functional avenues of these genes.

## Discussion

### Hints of Compensatory Mechanisms in Psychological Stress Manifestation

Enrichment analysis of **II(S)
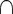
M(S)** with **II
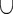
M** as background revealed that most of the enriched terms were populated by the following genes: IL6, IL1B, IL10, IL13, TNF and IFNG. These are the genes which are involved in the inflammatory response. Owing to the very high enrichment of the inflammatory cytokines, the data thus hints at the association of inflammatory response with psychological stress manifested in the body, as has already been observed and reported in the literature. Critical to this observation is the fact that while IL6, IL1B, TNF and IFNG are pro-inflammatory cytokines generally involved in acute inflammatory response, IL10 and IL13 are believed to be anti-inflammatory cytokines. This would suggest that both pro-inflammatory and anti-inflammatory cytokines are associated with the stress response (as is also justified by the enrichment of GSK3 in cluster 4 of the II(S)
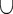
M(S) network [49]), which seems contradictory. But, this observation is quite relevant in the context of psychological stress since, pro-inflammatory cytokines are produced in response to a psychological stressor, but if they continue to be produced, they can have a damaging effect on the body which includes septic shock. Therefore, the body must counter-balance the effects of pro-inflammatory cytokines by inducing an anti-inflammatory response. The enriched GO terms also show the up-regulation of protein secretion and transport in addition to regulation of the acute inflammatory response as would be quite expected during body’s inflammatory responses. It is already known that CARS (Compensatory Anti-inflammatory Syndrome) develops in response to SIRS (Systemic Inflammatory Response Syndrome) to protect the body from the harmful effects of pro-inflammatory cytokines and acute phase proteins to restore the basal homeostasis of the body. In the case of psychological stress, it seems quite logical that the body’s adaptive response follows the same rule as can also be observed from the pattern of enriched GO terms in the enrichment analysis.

### Role of the Endo-Cannabinoid System (ECS)

As mentioned earlier, the CNR1 gene is consistently present in most of the enriched terms in the **II(S)
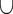
M(S)** network, which already hints towards direct or indirect association of this gene with psychological stress. This gene is one of two components of the Endo-Cannabinoid System, the other component being CNR2 (though not present in the enriched gene ontology terms). Interestingly, a review of the relevant literature revealed the fact that the CNR1 gene is associated with systemic homeostasis and can be considered a potential candidate bridging the stress response with energy balance, as it reportedly gets activated after stress as one of the recovery mechanisms [50]. Interestingly, the enrichment data shows clear absence of any association of these genes in the **II
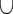
M** network since, when only “innate immunity” or “metabolism” is used as the keyword, there were few reports that relate the CNR1 or CNR2 gene with them indicating that the ECS would be inactive in physiological conditions. While, when “psychological stress” is used as a key-word the genes of this system represent the enriched fraction of the total genes pulled out indicating that ECS is active only in physiologic conditions associated with stress. Cannabinoid receptors are expressed mainly in the brain (central receptors), but some of them are also present in organs involved in energy homeostasis like adipose tissue, liver, gastro-intestinal tract, pancreas, skeletal muscle, etc (peripheral receptors). The endo-cannabinoid system has a dual mode of action on metabolism; activation of central cannabinoid receptors is believed to increase satiety which may lead to obesity, thereby promoting food energy efficiency, while activation of peripheral cannabinoid receptors (adipose tissue, liver, etc) can regulate metabolism and lipid storage without increasing food intake by some unknown mechanism [51], [52]. Reports also indicate that long-term effects of endo-cannabinoid system on metabolism could primarily be due to peripheral cannabinoid receptors [51]. This analysis implies that if the ECS is activated for longer time frames, it might cause food-intake independent weight gain (i.e. increased energy efficiency), which ideally fits within a context of chronic psychological stress. Reported observations thus lean towards the hypothesis that during stress, when the body requires instantaneous energy, this system might be dormant, but post-trauma the body tries to compensate for the energy expenditure during stress by increasing the activity of the ECS, thereby promoting storage of fats causing unnecessary energy conservation. An intriguing question to address here would be whether the increase in production of endo-cannabinoid ligands or expression of endo-cannabinoid receptors is responsible for post trauma enhanced activity of the ECS. Acute stress activates systems which require more energy, while chronic stress might lead to a constant over-activation of the ECS, leading to the metabolic syndromes like excessive weight gain, insulin resistance and dyslipidemia [53].

### Involvement of the Serotonin Receptor in Energy Homeostasis

The HTR1B and HTR2A genes have been consistently present in most of the enriched terms in the **II(S)
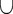
M(S)** network, thereby hinting at the association of 5-HT receptors (5-Hydroxytryptamine receptors) to psychological stress. 5-HT receptors are implicated in the regulation of feeding behavior, mood and even temperature regulation. Interestingly, in cluster 4 of the **II(S)
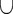
M(S)** network (Table S3 and figure 6), the sub-cluster formed by the 5-HT receptor family genes (marked as ‘**b**’) is linked to the sub-cluster of inflammatory cytokines (marked as ‘**a**’) via the POMC gene. It is already clear from previous discussions that psychological stress seems to induce a systemic inflammatory state in the body as is also justified by the enriched clusters of inflammatory cytokines. Inflammation is an energy intensive process [54] and sickness syndrome (which also includes elevation of body temperature) can be induced in the body to provide for this energy demand, since it is one of the energy conservation strategies of the body. Contrariwise, digestion is also an energy demanding process and so, it is quite expected that feeding behavior and digestion would be inhibited during systemic inflammation. The serotonin receptor HTR1A is known to induce hyperphagia [55], while HTR1B and HTR2C are known to reduce food intake [56] [46]. This kind of enrichment again appears contradictory, but here it should be noticed that this is an association study and should not be analyzed in the same manner as in co-expression studies. Enrichment of HTR1A, HTR1B and HTR2C in the same cluster indicates their association with POMC and with each other in different studies (i.e. quite possibly in different physiological conditions), and does not necessarily need them to be co-expressed under the same situation. While HTR2C and HR1B might contribute to the anorexigenic effect of the body during the inflammatory stage, HTR1A might work in conjunction with CNR1 to increase appetite during an adaptive response as a compensatory mechanism for energy balance. Another effect of HTR1A is induction of various hormones including cortisol, corticosterone, ACTH, oxytocin and B-endorphin. Cortisol and corticosterone are known to suppress the pro-inflammatory cytokines (thereby causing immune suppression) [57], while B-endorphin contributes to anti-depressant effects, thereby controlling inflammation and enabling the body to revert back to basal levels of metabolism. There are different reported mechanisms by which HTR1A causes activation of CRH, followed by activation of ACTH, finally inducing secretion of cortisol [58]. As has already been pointed out, many other proteins which might be acting during the compensatory response viz. CNR1 might also contribute to the activation of CRH and thereby lead to cortical/corticosterone production. Now, if the compensatory phase stretches beyond certain physiological limits, which might be the case in chronic stress, there could be a continuous over-activation of the ECS, serotonin receptor signaling and many more process which could have a possible role in the post-stress recovery system [59]. This could then cause obesity, dyslipidemia, insulin resistance and many more metabolic syndromes as well as a state of reduced immune resistance [54].

### The Feed-back Loop in Chronic Stress

Production of cortisol or corticosterone suppresses inflammatory cytokine responses and mobilizes glucose out of the cells, while high levels of glucose is known to induce production of IL6 (a pro-inflammatory cytokine) from monocytes. Even acute hyperglycemia in non-diabetics has been reported to elevate plasma IL6 and TNFα concentrations [60]. The data indicate that the glucose level in plasma is elevated during psychological stress as an effect of the HPA activity by corticosteroids. It has been reported that an inflammatory condition is created in the body when the plasma glucose level rises beyond a certain limit. The likelihood of this inflammation occurrence increases under hyper-corticoidism or a chronic stress situation [61]. This inflammatory condition could then again lead to activation of compensatory phase response which would now stretch beyond its normal range due to frequent induction (priming). The consequence of such an extended response might lead to obesity and many other associated metabolic syndromes [62].

**Figure 7:**
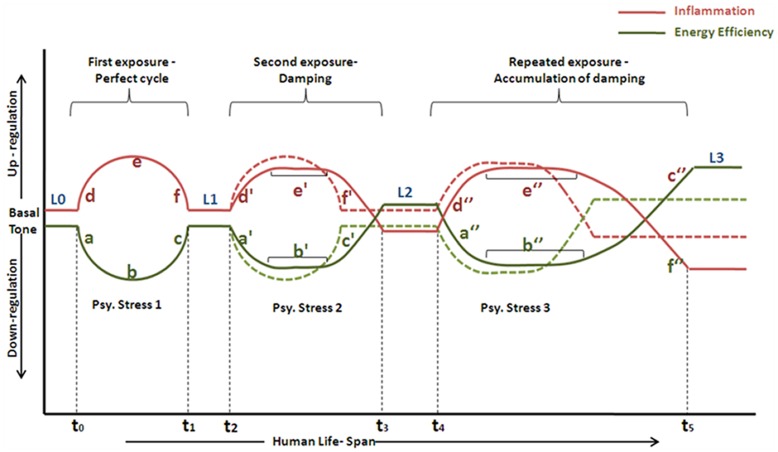
Meta-analysis based prediction model of psychological stress and associated homeostatic imbalance. A hypothetical model is developed to establish coping and adaptive responses to understand psychological stress as a challenge to normal physiology. Colored solid lines indicate the actual deviations from the basal tone, colored dotted lines represent expected, non-deviated basal tone after each cycle, while colored dashed lines show actual deviations from basal tone in case of a psychological stress challenge extending over a long time-period.

Based on the above observations and discussions, a hypothetical model has been proposed for explaining the interplay between various biological processes going on inside the body when a body is subjected to psychological stress and its manifestations on the body in long run (Figure 7). Basal tone of inflammatory state and energy efficiency in terms of body mass index are represented by L0. In the presence of psychological stress, inflammatory response is evoked (shown as ‘d’) by activation of innate defense responses and increase in production of pro-inflammatory cytokines. At the same time, a sickness behavior [63] is induced in the body which cuts down all energy expensive processes including food intake and digestion. This sickness situation can raise body temperature and can make the body socially withdrawn to conserve energy. The initial phase of such behavior is dominated by production of flight-fright hormones (catecholamines) which trigger lipolysis, glycogenolysis [64]. All these processes are aimed at providing the energy required for maintenance of the high inflammatory state by reducing the energy efficiency in terms of body mass (shown by ‘a’). Flight- fright response is followed by an increase in the production of cortisol which tends to raise the glucose level in blood by stimulating catabolic processes. Rising cortisol has inhibitory effects on the production of pro-inflammatory cytokines, which leads to a brake on the increasing slope of inflammatory state (only after attaining the threshold concentrations in blood) to reach a plateau (‘e’) and then declines (‘f’). The ECS and other stress recovery systems get activated by some unknown mechanism(s), which increase appetite, reduce the sickness behavior and increase energy efficiency by inducing weight gain (‘c’) to bring the body back to its basal tone (L1). However, the reasons, to initiate the transition from ‘b’ to ‘c’ and consequently to activate ECS and other stress recovery systems, are yet to be resolved. The proposed model (figure 7) suggests that extremely high levels of cortisol in blood during ‘b’ might trigger the activation of stress recovery systems and initiate transition from ‘b’ to ‘c’. This transition also indicates the condition where cortisol levels start declining and reach basal levels (L1) by time t_1_. It is important to note that excess of glucose levels are known to increase production of pro-inflammatory cytokines [65], [66]. Blood glucose levels might be one of the mechanisms involved in controlling cortisol levels (since excess of cortisol can create a hyperglycemic state). Proposed model seems fitting to explain the mechanisms of restoring homeostasis when perturbed by psychological stress. The scenario, however, may not remain simple if the physiology is repeatedly experiencing challenges such as psychological stressor or if the challenge itself persistently lasts extending over a significantly longer period. Figure 7 reveals that an individual when encounters the challenge for second time, the slope of increasing inflammatory state (d’) and decreasing energy efficiency (a’) decreases. This observation suggests that the physiological response following the challenge is slower. The plateau stage also probably extends for a longer time (b’), which implies that cortisol acts for a longer period and hence suppresses the inflammatory cytokines below the basal levels (f’). In this situation, body glucose levels would not be able to regulate cortisol levels by feed-back mechanisms, since most probably the stress recovery systems which act longer now (c’) use up the excess blood glucose for anabolic activities for increasing energy efficiency. Thus, by time t_4_ or at longer interval depending on the individual physiology both these process reset the physiological basal tone at L2, which denotes a higher energy efficiency in terms of body mass, and a lower than normal inflammatory state. Again when the body encounters the challenge a third time, the basal tone becomes reset at a much deviated level, L3 by time t_6_. Thus with each cycle, a damping effect on these systems is most likely to be exhibited if they are frequently stimulated. This dampening effect causes the compensatory mechanisms to deviate from the basal tone and become reset such that the body weight is increased and the innate immune responses are lowered down.

The hypothetical model proposed is based on various experimental data reported in the literature. While development of a probabilistic model would lead to more elaborative detailed studies and certainly would be beyond scope of the current report, we have suggested a general empirical mathematical model to explain the model proposed in this report. The relationship of action and counter actions to maintain homeostasis is following a rule which can be formulated as.



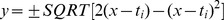



Where, ‘y’ is the effect and ‘x’ is the cause and ‘t_i_’ is time interval of experiencing the cause at different interval ‘i’. In the current model, at i = 0 value of ‘t_o_’ is zero and the first phase cycle ends at i = 1 or at t_1,_ similarly, second phase of stress, if experienced, starts at t_2_ it will end at t_3_ and so on until the effects of stress experience becomes long enough in terms of time interval so that it is considered as stress invariant or in other words defined as chronic stress. In the current equation for any phase, effects on perturbation and restoration of homeostasis will have three time intervals. For example, at (a) t_o_, x ranges between 0 and 1, (b) x = 1 for x ranges between 1 and 1+t_o_ and (c) x ranges between 1+t_0_ and t_1_ ( = 2+t_o_). For subsequent phases, time interval will be longer and determining the stage for an individual to transform from acute phase to chronic. Sign (±) of the final effect will determine upward to downward effects based on the physiological consequences.

Physiologically, this mechanism has the potential to explain the reason which might increase susceptibility to infection and might lead to obesity and other metabolic syndromes. However, detailed analysis and studies are yet to be established and beyond scope of the current report. An important point to note here is that body’s adaptive response might also depend on the interval between two challenges viz. t_1_−t_2_ and t_4_−t_5_, which might also decide where the body’s basal tones are being reset after each cycle. However, in the case of a challenge which extends over a long time-period t_1_−t_3_ (shown by dashed lines), where cortisol acts longer and thereby suppresses the inflammatory state below the basal tone, while the compensatory stress recovery system works longer in response to it and the body’s basal tone becomes rest at L1’ instead of L1 at time t_3_. This suggests a justified explanation for infectious disease susceptibility [1], [67], obesity [68] and other metabolic syndromes associated with chronically stressed people. This also hints to the aberrant weight loss, withdrawn behavior and flu-like symptoms (due to acute phase response) seen in people have short-term acute stress shown by the initial fluctuations from basal tone when body encounters psychological stress. Based on this model, physiological homeostasis of our body can assumed as an elastic system, which rebounds to its original shape after the stress stimuli is withdrawn. But when the body tries to push beyond the elastic limits of its adaptive scope, a hysteresis loss (biologically exemplified by mal-adaptation/dysfunction) is expected, which is well supported by the analysis based on observations so far in literature related to psychological stress.

## Limitations

The mechanisms suggested here are based on systematic review which is dependent on studies which have already been done and reported in the literature. It is not ruled out that studies may not have been attempted in many areas which might have important implications to the understanding of psychological stress and the associated physiology. On the contrary, it is also possible that due to extensive studies on select biological processes, certain genes might be over-represented. These can definitely induce a bias in an analysis such as the one presented here, but our approach was designed to minimize such bias by introducing special strategies of data refinement. Experimental cross-validation of these suggestive mechanisms would be helpful in understanding the molecular signaling of these events which could aid in designing intervention techniques [69] to tackle the numerous manifestations of psychological stress.

## Supporting Information

Checklist S1 **PRISMA Checklist.**
(DOC)

PSI-MI Data S1 **Proteomics Standards Initiative (PSI)-Molecular Interactions (MI) data for all cytoscape clusters and interactions data are shown in xml format.**
(ZIP)

Schema S1 **PRISMA flowchart for study selection, screening and inclusion.**
(TIF)

Table S1 **Training set genes obtained through manual curation.**
(DOC)

Table S2 **The candidate test-set genes obtained from the training set using ENDEAVOUR.**
(DOC)

Table S3 **Enrichment details of the consensus enriched clusters.**
(DOC)

Table S4
**Comparison of the genes of II(S)∩M(S) against II
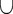
M network.**
(DOC)

Table S5 **The total number of references pulled out by AGILENT literature search plugin for Metabolism network.**
(TXT)

Table S6 **The total number of references pulled out by AGILENT literature search plugin for Innate immunity network.**
(TXT)

Table S7 **The total number of references pulled out by AGILENT literature search plugin for Metabolism & PS network.**
(TXT)

Table S8 **The total number of references pulled out by AGILENT literature search plugin for INNATE immunity & PS network.**
(TXT)

Table S9 **Data statistics for Node and Edge data presented in Tables S5–S8.**
(XLS)

Table S10 **Reference IDs for data statistics presented in Table S9.**
(XLS)
