# Supplementary figures and images for: Effects of Psychological Stress on Innate Immunity and Metabolism in Humans: A Systematic Analysis
Source: PLoS One. 2012 Sep 19;7(9):e43232. doi: 10.1371/journal.pone.0043232 (PMC3446986; doi:10.1371/journal.pone.0043232)

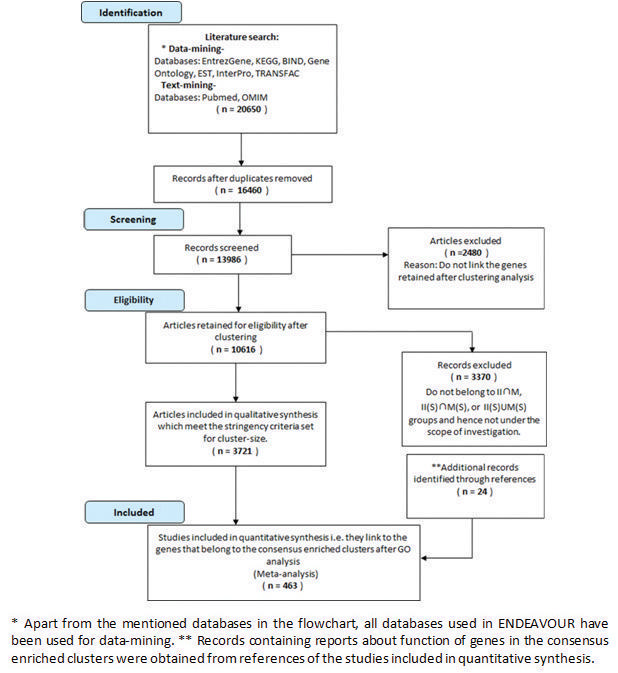

Supplement: Schema S1 — PRISMA flowchart for study selection, screening and inclusion. (TIF) [file pone.0043232.s003.tif]
