# Supplementary material for: Effects of Psychological Stress on Innate Immunity and Metabolism in Humans: A Systematic Analysis
Source: PLoS One. 2012 Sep 19;7(9):e43232. doi: 10.1371/journal.pone.0043232 (PMC3446986; doi:10.1371/journal.pone.0043232)
Supplement: Table S1 — Training set genes obtained through manual curation. (DOC) [file pone.0043232.s004.doc]

**Table S1:** Training set genes obtained through manual curation.

| **Innate-Immune Training-set genes** | | **Metabolism Training-set genes** | | | |
| --- | --- | --- | --- | --- | --- |
| CD14  IL16  IL6  NOD2  IFIH1  NLRC4  DDX58  IFNA2  IFNB1  TBK1  ABL1  IFNG  TICAM1  TLR2  TLR4  CFH  IL4R  TNF | CASP8  TGFB1  PTPN22  IL23R  IRF5  CTLA4  IRF7  IRF3  TRAF5  EYA4  NLRX1  PSMA7  UCP2  CCL4  CCL5  MYD88  BAX  AIM2 | HNF4  PRARA  GCK  INSR  VDR  IRS2  ADIPOQ  INS  PTEN  PTENP1  TCF2  SLC2A2  CP  IFNG  RXRG  GYS1  CPE  NOS3  TCF1  UCP3  IRS1  CSH1  GH1  NEUROD1 | LIPE  PDX1  STX1A  UCP1  TAP2  LMNA  HP  BCHE  ABCC8  IGF1R  WFS1  RXRA  RETN  ACE  THRA  SAA1  ATP2A3  SAA4  KCNJ11  IAPP  SORBS1  CD38  CTLA4  NOS2A | PTGS2  PGR  GPD2  DCN  AVP  AVPR2  NR1H3  RARA  PPARD  EP300  UCP2  RARB  ONECUT1  AR  GCKR  FOXA2  PGDFRB  LEP  THRB  TGFB1  CRP  NCOA3  GHR  KCNJ9 | FABP4  ALB  APCS  NR2F1  AGRP  ESR1  NOS1  KCN8  APOA2  ANG  RNASE4  TCF7  FN1  AKT1  RARG  TITF1  GYS2  AHSG  SOCS3  HK1  CFH  FOXO3A  LPL  NR0B2  PPARG |
