# Supplementary material for: Effects of Psychological Stress on Innate Immunity and Metabolism in Humans: A Systematic Analysis
Source: PLoS One. 2012 Sep 19;7(9):e43232. doi: 10.1371/journal.pone.0043232 (PMC3446986; doi:10.1371/journal.pone.0043232)
Supplement: Table S2 — The candidate test-set genes obtained from the training set using ENDEAVOUR. (DOC) [file pone.0043232.s005.doc]

**Table S2:** The candidate test-set genes obtained from the training set using ENDEAVOUR.

| **Innate Immunity Candidate-Set genes** | | | | | | | | | |
| --- | --- | --- | --- | --- | --- | --- | --- | --- | --- |
| MYD88 | CCL4 | TLR8 | BID | TRAF3 | PSMA4 | IL7R | PTPRC | NCOA3 | PSMA6 |
| TLR2 | TLR6 | IFNAR2 | CASP6 | IL6R | BTK | PPBP | CFH | IRAK3 | TNFAIP3 |
| IRF7 | TLR5 | CXCL10 | TNFRSF1A | PSMA2 | JAK1 | STAT6 | FLT3 | CASP4 | CASP2 |
| IL6 | TLR1 | PSMA7 | IL1R1 | IFNA1 | TLR10 | TGFBR2 | ZAP70 | CD86 | IGF1R |
| TLR4 | CCL3 | CCL8 | CD40 | IL1B | IRAK1 | TGFB3 | NLRC4 | CCL16 | RAF1 |
| CCL5 | CASP1 | IFIH1 | TIRAP | IKBKG | HCK | APAF1 | IFNA2 | STK4 | BIRC3 |
| IRF3 | PTPN22 | TICAM1 | IFI16 | IRF1 | CCL1 | IFNA17 | CCL26 | IFNA5 | NLRP3 |
| IL4R | DDX58 | TLR7 | ABL2 | MAP3K3 | TRADD | CSF1R | AKT1 | FASLG | PRKCD |
| TNF | CFLAR | IKBKE | IL1RAP | IFNW1 | CD28 | MAP3K7 | TANK | IL8 | RAF1 |
| IFNG | NOD1 | CCL7 | IFNGR1 | PSMA5 | IFNA10 | IFNA4 | EIF2AK2 | IRF8 | TLR9|TWF2 |
| CASP8 | IL16 | FAS | CCL13 | MAP3K1 | TYK2 | FLNA | CCL3L3 | CEBPB | CCL14|CCL15 |
| TBK1 | IRF5 | IL23R | CSF2RB | CSK | MAP3K8 | CARD8 | CD180 | IL2 | CHUK|ERLIN1 |
| BAX | IL4 | CD80 | CXCL11 | CXCL9 | NFKB2 | IL18RAP | CASP9 | UCP3 | ISGF3G|RNF31 |
| TRAF5 | LCK | RELA | STAT1 | SYK | BAK1 | IL18R1 | CXCR4 | CCL18 | BIRC3TNFRSF10A |
| IFNB1 | UCP2 | TNFSF10 | LY96 | CARD11 | BCL10 | CCL3L1 | TNFSF8 | NLRP3 | TNFSF12|TNFSF13 |
| NOD2 | IL2RG | RIPK1 | CCL17 | TGFB2 | LILRB4 | ITGB2 | ITGAL | PRKCD | ENSG00000115607 |
| ABL1 | CASP10 | FADD | IL2RB | NFKB1 | GRAP2 | IFNA13 | TNFRSF11A | RAF1 | ENSG00000206439 |
| TGFB1 | RIPK2 | IKBKB | LTA | TRAF2 | NFKBIA | BCL2A1 | BCL2L1 | BIRC3 | ENSG00000206328 |
| CTLA4 | BIRC2 | CASP3 | CCL11 | MAPK14 | NLRP1 | LYN | IRAK4 | IRF2 | ENSG00000000971 |
| CD14 | CCL2 | TNFRSF1B | IRF4 | IRAK2 | TRAF1 | VAV1 | OSMR | NLRP3 |  |
| TLR3 | TRAF6 | IL6ST | IFNGR2 | IL23A | HCLS1 | JAK2 | TNFRSF8 | PRKCD |  |

| **Metabolism Candidate-Set genes** | | | | | | | | | |
| --- | --- | --- | --- | --- | --- | --- | --- | --- | --- |
| HNF4 | NOS1 | TSC2 | FABP3 | ADRB2 | DRD2 | RXRA | ADCY2 | LDHA | PRKCD |
| PRARA | KCN8 | NR1H4 | KCNJ3 | AGTR1 | GNB3 | PGR | LRP2 | TYK2 | NR5A2 |
| GCK | APOA2 | SYN1 | APP | DRD1 | LCAT | TRHR | CHRNA7 | EDG7 | NCOA3 |
| INSR | ANG | FABP1 | STAT1 | LPL | APOA2 | NR5A1 | GRM1 | HRH1 | PRKCA |
| VDR | RNASE4 | EGFR | JAK1 | APOE | IGFIR | DRD5 | ESRRG | PLTP | NR1I3 |
| IRS2 | TCF7 | NR2F6 | F9 | NR3C1 | LIPC | GNAI2 | MERTK | CNR1 | PAPPA |
| INS | FN1 | COL7A1 | PRLR | GNAS | EGFR | CHRM3 | CREBBP | EDG1 | GNA11 |
| PTEN | AKT1 | CEBPB | IRS4 | GHR | INSR | EP300 | ADCY6 | APOC1 | NPY1R |
| TCF2 | RARG | BRAF | FGFR3 | IRS1 | HNF4A | PPARD | TBXA2R | MC2R | MYH10 |
| CP | TITF1 | LCK | FOXA1 | PPARG | APOL1 | ERBB2 | FASN | CFH | PCDH1 |
| IFNG | GYS2 | MAPK14 | LYN | CETP | PON1 | PTPRC | AVPR1B | PTHR1 | PTGDR |
| RXRG | AHSG | SUMO4 | AGTR1 | CAT | NR2F6 | CAV1 | NR2F1 | FYN | MC4R |
| GYS1 | SOCS3 | CFTR | STAT5B | AGT | ESR1 | HTR | ACSM3 | CHRM5 | PTENP1 |
| CPE | HK1 | ERBB2 | AKT2 | ABCA1 | THRA | CXCR4 | AKT1 | LEP | SLC12A4 |
| NOS3 | CFH | GSK3B | NR1D1 | BDKRB2 | NPR2 | EDN1 | FLNA | FLT4 | TNFRSF1A |
| TCF1 | LPL | CAPN10 | POU2F2 | NOS3 | TSHR | MAS1 | RARB | HTR2B | SERPINC1 |
| UCP3 | NR0B2 | APOA1 | G6PC | SCNN1A | RETN | FGFR3 | PTGIS | VIPR1 | ADORA2A |
| IRS1 | ESR2 | NR1I2 | GUSB | LEF1 | OPRM1 | DRD3 | ACE | CD36 | ADRA2A |
| CSH1 | CREBBP | NR4A2 | MPO | SCARB1 | ERBB3 | CHRM2 | CASP6 | LRP1 | CACNA1C |
| GH1 | CSK | TAP1 | TGFB1 | SCNN1B | THRB | AVPR2 | LCK | NPPC | ADORA2B |
| PPARG | ABCC2 | VAV2 | HSD3B1 | TNF | PRLR | OPRK1 | PPARA | HTR1F | CSH1|GH1 |
| LIPE | ESRRG | PTPN11 | FN1 | EDNRA | APOA4 | ERB4 | CSK | NR1D1 | NEUROD1 |
| PDX1 | RXRB | VAV1 | TCF7L1 | NR3C2 | NR1H4 | HTR6 | IKBKB | OPRL1 | ONECUT1 |
| STX1A | PTPRC | ALDOA | CRHR1 | ADM | OPRD1 | FGFR3 | NR3C1 | DDR1 | CACNA1A |
| UCP1 | GIPR | PAX6 | MET | LIPG | GNAQQ | P2RY1 | ADRA1A | F2RL1 | SLC25A13 |
| TAP2 | PIK3R1 | SHC1 | TGFB1 | APOC3 | PDGFRA | HRH2 | CYP11A1 | OXTR | SERPIND1 |
| LMNA | ENPP1 | SP1 | PTPN1 | VDR | CCKBR | AGTR2 | AVPR1A | APOC4 | MAPK8IP1HK2 |
| HP | SLC2A1 | TP53 | PAX4 | ADRB1 | ESR2 | DRD1IP | HSD11B2 | SSTR2 | ENSG00000206218 |
| BCHE | IGFBP3 | IFNGR1 | ADRA1B | NPR1 | CHRM1 | MET | CACNA1D | ADRBK1 | ENSG00000206289 |
| ABCC8 | ERBB3 | IGFBP6 | PLG | ADRB3 | NR1I2 | TEK | CYP11B1 | FOXO1A | ENSG00000166285 |
| IGF1R | LHX3 | IGF1 | DRD2 | NPR3 | SCNN1D | AHSG | SCNN1G | FOXO3A | ENSG00000206439 |
| WFS1 | FOXP3 | ATP2A2 | CD86 | APOC2 | GH2 | HRH4 | LHCGR | ADIPOQ | ENSG00000206328 |
| RXRA | SLC2A4 | ABCC6 | APOC3 | NR1H3 | FGB | ALB | CYP11B2 | F2R | ENSG00000206299 |
| RETN | FLNA | ADRB2 | ADRB3 | EDNRB | NR0B2 | LBP | ADRA1B | TCF7L2 | ENSG00000206235 |
| ACE | CD80 | FGFR1 | INSL3 | ADD1 | NOS1 | FSHR | ADORA1 | EDN2 | C14orf2|LTBR4| LTB4R2 |
| THRA | CYP1A1 | APOA1 | JAK3 | APOB | PDGFRA | NR41 | PGDFRB | SLC2A2 |  |
