# Supplementary material for: Effects of Psychological Stress on Innate Immunity and Metabolism in Humans: A Systematic Analysis
Source: PLoS One. 2012 Sep 19;7(9):e43232. doi: 10.1371/journal.pone.0043232 (PMC3446986; doi:10.1371/journal.pone.0043232)
Supplement: Table S3 — Enrichment details of the consensus enriched clusters. (DOC) [file pone.0043232.s006.doc]

**Table S3:** Enrichment details of the consensus enriched clusters

| **S.No** | **Network and Cluster Name** | **Cluster** | **Genes Involved** | **GO terms enriched** |
| --- | --- | --- | --- | --- |
| 1. | **Target:**  II∩M  **Background:**  IIM  Cluster 4 | 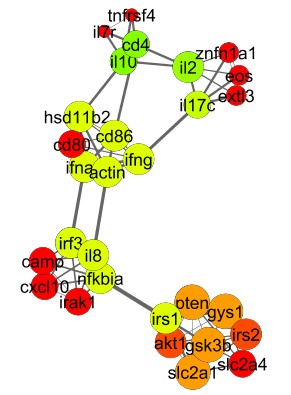 | actin, irs2, irak1, cxcl10, nfkbia, slc2a4, cd80, extl3, tnfrsf4, il2, ifng, znfn1a1, gys1, cd86, hsd11b2, irs1, pten, il8, akt1, gsk3b, slc2a1, il17c, il7r, il10, irf3, ifna1, camp, cd4, eos | -Regulation of protein secretion  - Positive regulation of cell activation  - Regulation of Immune response  - Positive regulation of leukocyte activation  -Positive regulation of lymphocyte activation  -Positive regulation of lipid metabolic process  - Regulation of fatty-acid B-oxidation |
| 2. | **Target:**  II∩M  **Background:** IIM  Cluster 10 | 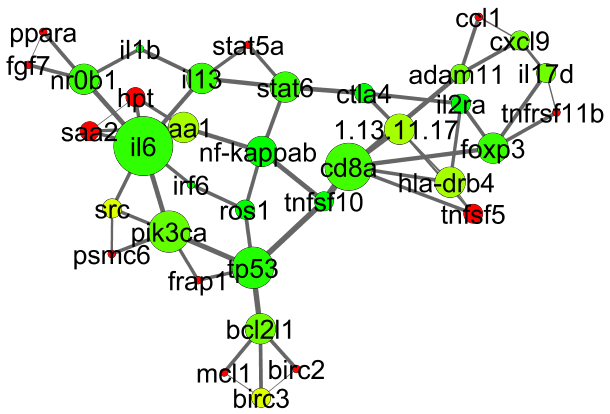 | cxcl9, hpt, saa1, adam11, hla-drb4, 1.13.11.17, irf6, nf-kappab, ros1, tp53, ppara, il17d, cd8a, il6, stat6, saa2, il13, ctla4, il1b, birc3, foxp3, smc6, fgf7, bcl2l1, frap1, tnfrsf11b, pik3ca, il2ra, src, ccl1, mcl1, nr0b1, birc2, tnfsf10, tnfsf5, stat5a | -Regulation of protein secretion  - Positive regulation of cell activation  - Regulation of Immune response  - Positive regulation of leukocyte activation  - Positive regulation of lymphocyte activation  - Regulation of lipid storage  - Regulation of cholesterol storage  - Positive regulation of lipid metabolic process  -Regulation of fatty-acid B-oxidation |
| 3. | **Target:**  II∩M  **Background:** IIM  Cluster1 | 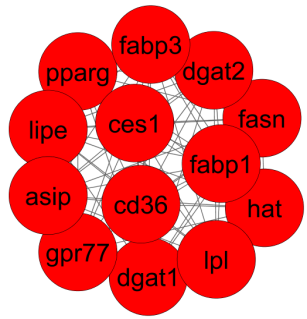 | asip, lipe, lpl, pparg, fasn, dgat1, fabp3  gpr77, cd36, ces1  dgat2, hat, fabp1 | -Regulation of Immune response  - Positive regulation of leukocyte activation  - Positive regulation of lymphocyte activation  - Regulation of lipid storage  - Regulation of cholesterol storage  - Positive regulation of lipid metabolic process  - Regulation of fatty-acid B-oxidation |
| 4. | **Target:**  II∩M  **Background:** IIM  Cluster8 | 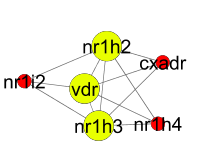 | nr1h2, nr1i2, vdr, nr1h4, nr1h3, cxadr | -Regulation of protein secretion  -Positive regulation of cell activation  -Regulation of Immune response  -Positive regulation of leukocyte activation  - Positive regulation of lymphocyte activation  - Regulation of lipid storage  -Regulation of cholesterol storage  -Positive regulation of lipid metabolic process |
| 5. | **Target:**  II(S) M(S)  **Background:**  IIM  Cluster1 | 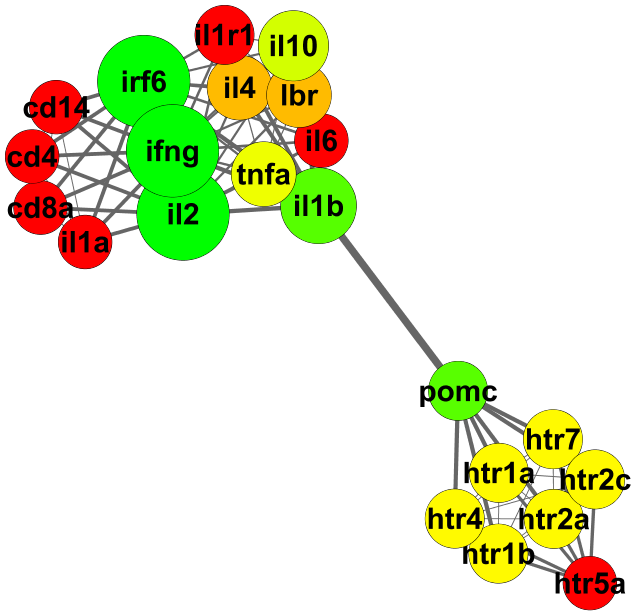 | htr5a, il1b, htr1b,  il2, htr2c, il10,  pomc, il1a, htr4,  il1r1, ifng, htr7,  irf6, cd4, htr2a  tnfα, cd14, cd8a  il4, lbr, htr1a, il6 | -Regulation of systems process  - Regulation of systems neurological process  - Negative regulation of multicellular organismal process  - Positive regulation of protein secretion  - Regulation of immunoglobin  - Response to temperature stimulus  - Behaviour  -G-protein coupled receptor protein signaling pathway |
| 6. | **Target:**  II(S) M(S)  **Background:**  IIM  Cluster2 | 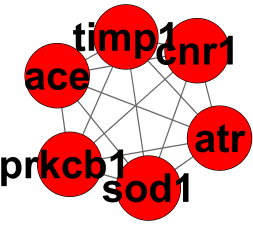 | Ace, sod1, cnr1, timp1, prkcb1,  atr | -Regulation of systems process  -Regulation of systems neurological process  -Negative regulation of multicellular organismal process  -Positive regulation of protein secretion  -Regulation of immunoglobin  -Response to temperature stimulus  -Behaviour  -G-protein coupled receptor protein signaling pathway |
| 7. | **Target:**  II(S) M(S)  **Background:**  IIM  Cluster4 | 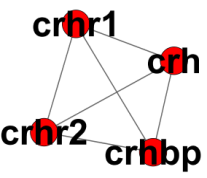 | Crh, crhr1, crhr2,  crhbp | -Regulation of systems process  - Regulation of systems neurological process  - Negative regulation of multicellular organismal process  - Positive regulation of protein secretion  -Regulation of immunoglobin  - Response to temperature stimulus  - Behaviour  -G-protein coupled receptor protein signaling pathway |

This table contains details of the consensus clusters ( consistently populated by majority of the significantly enriched GO terms) in the enrichment analysis of genes populating II∩M and II(S)∩M(S) networks against a background of IIM network; where a minimum of 10^-7^ enrichment p-value is set as limit of significance for our analysis. The clusters have been named according to their original ranks in MCODE clustering analysis.
