# Supplementary material for: Effects of Psychological Stress on Innate Immunity and Metabolism in Humans: A Systematic Analysis
Source: PLoS One. 2012 Sep 19;7(9):e43232. doi: 10.1371/journal.pone.0043232 (PMC3446986; doi:10.1371/journal.pone.0043232)
Supplement: Table S4 — Comparison of the genes of II(S)∩M(S) against IIM network. (DOC) [file pone.0043232.s007.doc]

**Table S4:** Comparison of the genes of II(S)∩M(S) against IIM network.

| GO Term | Description | P-value | Enrichment  (N, B, n, b) | Genes |
| --- | --- | --- | --- | --- |
| GO:0002637 | regulation of immunoglobulin production | 2.47E-07 | 17.46 (774,14,19,6) | IL13 - interleukin 13  IL6 - interleukin 6 (interferon, beta 2)  IL10 - interleukin 10  TNF - tumor necrosis factor  IL5 - interleukin 5 (colony-stimulating factor, eosinophil)  IFNG - interferon, gamma |
| GO:0045428 | regulation of nitric oxide biosynthetic process | 6.41E-07 | 15.28 (774,16,19,6) | PTGS2 - prostaglandin-endoperoxide synthase 2 (prostaglandin g/h synthase and cyclooxygenase)  IL6 - interleukin 6 (interferon, beta 2)  IL10 - interleukin 10  TNF - tumor necrosis factor  IL1B - interleukin 1, beta  IFNG - interferon, gamma |
| GO:0050714 | positive regulation of protein secretion | 6.41E-07 | 15.28 (774,16,19,6) | IL13 - interleukin 13  IL6 - interleukin 6 (interferon, beta 2)  IL10 - interleukin 10  TNF - tumor necrosis factor  IFNG - interferon, gamma  IL5 - interleukin 5 (colony-stimulating factor, eosinophil) |
| GO:0051043 | regulation of membrane protein ectodomain proteolysis | 1.29E-06 | 32.59 (774,5,19,4) | IL10 - interleukin 10  TNF - tumor necrosis factor  IL1B - interleukin 1, beta  IFNG - interferon, gamma |
| GO:0002697 | regulation of immune effector process | 2.07E-06 | 6.32 (774,58,19,9) | IL13 - interleukin 13  NOS2 - nitric oxide synthase 2, inducible  IL6 - interleukin 6 (interferon, beta 2)  IL10 - interleukin 10  TRAF3 - tnf receptor-associated factor 3  TNF - tumor necrosis factor  IL1B - interleukin 1, beta  IFNG - interferon, gamma  IL5 - interleukin 5 (colony-stimulating factor, eosinophil) |
| GO:0008285 | negative regulation of cell proliferation | 3.11E-06 | 4.48 (774,100,19,11) | PPARG - peroxisome proliferator-activated receptor gamma  APC - adenomatous polyposis coli  JUN - jun proto-oncogene  CYP27B1 - cytochrome p450, family 27, subfamily b, polypeptide 1  PTGS2 - prostaglandin-endoperoxide synthase 2 (prostaglandin g/h synthase and cyclooxygenase)  IL6 - interleukin 6 (interferon, beta 2)  IL10 - interleukin 10  TNF - tumor necrosis factor  IL1B - interleukin 1, beta  IFNG - interferon, gamma  IRF6 - interferon regulatory factor 6 |
| GO:0051222 | positive regulation of protein transport | 3.72E-06 | 8.91 (774,32,19,7) | IL13 - interleukin 13  IL6 - interleukin 6 (interferon, beta 2)  IL10 - interleukin 10  TNF - tumor necrosis factor  IL1B - interleukin 1, beta  IFNG - interferon, gamma  IL5-interleukin 5 (colony-stimulating factor, eosinophil) |
| GO:0060558 | regulation of calcidiol 1-monooxygenase activity | 3.80E-06 | 27.16 (774,6,19,4) | CYP27B1 - cytochrome p450, family 27, subfamily b, polypeptide 1  TNF - tumor necrosis factor  IL1B - interleukin 1, beta  IFNG - interferon, gamma |
| GO:0051047 | positive regulation of secretion | 4.66E-06 | 8.64 (774,33,19,7) | IL13 - interleukin 13  IL6 - interleukin 6 (interferon, beta 2)  IL10 - interleukin 10  TNF - tumor necrosis factor  IL1B - interleukin 1, beta  IFNG - interferon, gamma  IL5 - interleukin 5 (colony-stimulating factor, eosinophil) |
| GO:0050708 | regulation of protein secretion | 7.28E-06 | 10.63 (774,23,19,6) | IL13 - interleukin 13  IL6 - interleukin 6 (interferon, beta 2)  IL10 - interleukin 10  TNF - tumor necrosis factor  IL5 - interleukin 5 (colony-stimulating factor, eosinophil)  IFNG - interferon, gamma |
| GO:0002673 | regulation of acute inflammatory response | 8.87E-06 | 14.55 (774,14,19,5) | PPARG - peroxisome proliferator-activated receptor gamma  PTGS2 - prostaglandin-endoperoxide synthase 2 (prostaglandin g/h synthase and cyclooxygenase)  IL6 - interleukin 6 (interferon, beta 2)  IL1B - interleukin 1, beta  TNF - tumor necrosis factor |
| GO:0045429 | positive regulation of nitric oxide biosynthetic process | 8.87E-06 | 14.55 (774,14,19,5) | PTGS2 - prostaglandin-endoperoxide synthase 2 (prostaglandin g/h synthase and cyclooxygenase)  IL6 - interleukin 6 (interferon, beta 2)  TNF - tumor necrosis factor  IL1B - interleukin 1, beta  IFNG - interferon, gamma |

The enriched GO terms with a minimum enrichment p-value 10^-7^ have been included in the analysis. The pro-inflammatory cytokines (green and cyan) and anti-inflammatory cytokines (red and pink) have been colour coded for representing their distribution in the enriched GO terms.
